# Patient and health system determinants of experiences of care at primary health care clinics in eThekwini, KwaZulu-Natal, 2018

**DOI:** 10.4102/phcfm.v13i1.2884

**Published:** 2021-09-30

**Authors:** Avashri Harrichandparsad, Ozayr H. Mahomed

**Affiliations:** 1 Department of Public Health Medicine, University of KwaZulu-Natal, Durban, South Africa

**Keywords:** patients’ experience, primary health care clinics, health system, patient factors, ideal clinic

## Abstract

**Background:**

Respect for persons includes three sub-elements: dignity, autonomy and confidentiality, whilst client orientation has four sub-elements: prompt attention, quality of basic amenities, access to social support for hospitalised individuals and choice of health providers.

**Aim:**

This study sought to determine patient and health system determinants of experiences of care.

**Setting:**

Study was conducted at primary health care clinics in eThekwini, KwaZula-Natal.

**Methods:**

A self-administered questionnaire was used to collect data from 384 patients who received ambulatory care at six primary health care facilities (three community healthcare centres and three clinics) between June 2018 and November 2018.

**Results:**

Three hundred and sixty nine respondents were included in the study. Eighty one percent (299) of the respondents were female, 67.2% (248) were single and 89.7% (331) were black Africans. Fifty (13.6%) respondents reported their health status to be poor, whilst 47 (12.5%) reported excellent health, with the majority (72.0%) reporting ‘good’ or ‘fair’ health. The patients’ experience score for the study population was 89.0% (IQR 81% – 98%). Patients who attended clinics had a 6.53 (*p* < 0.001) times increased odds of reporting good patients’ experience score compared with patients who attended community healthcare centres. Although ideal clinic status had a positive association with patients’ experience score (odds ration [OR]: 1.75; *p* > 0.05) this was not significant.

**Conclusion:**

Patients attending clinics had a better experience compared with community health centres. Ideal clinic status showed a positive but not statistical significant association with good patient experiences. This may suggest that factors other than structural improvements play an important role in patients’ experience.

## Introduction

Universal Health Coverage (UHC) has emerged as the global priority intervention and the overarching goal of the 2015 post development agenda.^1^ Universal health coverage has ensured that health system strengthening is the main focus to improve the level and distribution of health and health services.^2^ South Africa, similar to other lower- and middle-income countries has commenced implementing the National Health Insurance to address the inequities of the healthcare system, achieve better health outcomes, improve responsiveness to the non-health needs of patients, offer financial risk protection and to increase efficiency.^3^

Responsiveness, although indirectly related to health, is seen as an intrinsic goal in its own right, which influences the quality of services delivered.^4^ The concept of responsiveness as defined by the World Health Organization has two components: respect for persons and client orientation.^5^ The dimension of responsiveness known as ‘respect for persons’ entails treating patients with dignity, involving them in decisions about their care, communicating clearly with them and maintaining confidentiality.^5^ Responsiveness also includes actions to ensure client-orientation, which include reduced waiting times, access to social support, choice of provider and basic amenities of adequate quality.^5^

Legislation, policies, strategies and guidelines aim to improve the quality of healthcare in South Africa. Section 27 of the Bill of Rights, enshrined in the Constitution of the Republic of South Africa (Act 108 of 1996)^6^ ensures the right to high quality responsive healthcare that the state must progressively realise. As a means to ensure the protection of human rights in the public health service, the Batho Pele White Paper on Transforming Public Service Delivery of 1997 set out eight guiding principles, namely consultation, service standards, access, courtesy, information, openness and transparency, redress and value for money.^7^ This key policy document gave rise to two further policy initiatives, namely the 2007 Patients’ Rights Charter and National Policy on Quality, which also emphasised responsiveness to the non-health needs of clients providing for increased patients participation and dignity afforded to them. In 2010, the National Department of Health launched its 10-point plan to improve the health system, which also emphasised actions aimed at responsiveness.^8^ Priority three was the improvement of the quality of health services through the development of National Core Standards for health establishments with specific reference to six priority areas, namely patient safety and security, infection prevention and control, cleanliness, availability of medicines, waiting times and positive and caring staff attitudes.^8^

The adoption of national core standards for healthcare facilities in South Africa in 2011 was a key initiative that addressed improvements in both efficiency and responsiveness. Domain one of the National Core Standards addressed health system responsiveness to the non-health needs of patients in emphasising patient-centred care and set standards for ensuring that patients’ rights, according to the Patients’ Rights Charter and Batho Pele Principles, were upheld.^9^ This domain specifically listed annual patient satisfaction surveys and their use in informing quality improvements as a minimum requirement and also addressed access to care and respectful, informed and dignified attention within a hygienic environment.^9^

**FIGURE 1:**
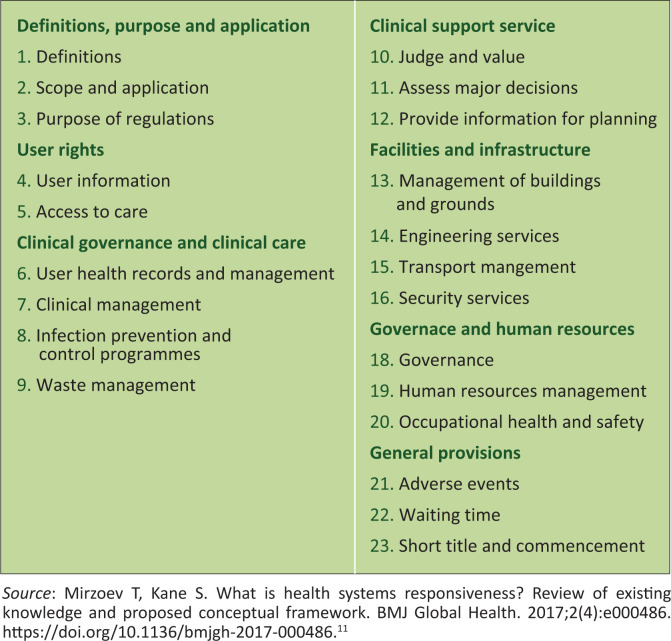
Sub-regulations as per Regulation 67: Norms standards and regulations applicable to different categories of health establishments.

On 2 February 2018, the National Minister published Regulation 67: Norms and Standards Regulations Applicable to Different Categories of Health Establishments to promote and protect the health and safety of users and healthcare personnel.^10^ These regulations contain 23 sub-regulations across the following domains: user rights, clinical governance and clinical care, clinical support services, facilities and infrastructure, governance and human resources and general provisions (Figure 1).^10^

The World Health Organization proposed seven elements against which responsiveness can be measured, namely dignity, autonomy, confidentiality, prompt attention, quality of amenities, access to social support networks and choice of service provider.^11^ It covers different aspects of individual’s satisfaction with medical and non-medical aspects of healthcare^12^ and focuses on self-assessment.

Considering the legislative imperatives and the ideal clinic elements of an annual patient experiences survey the National Department of Health provided a National Guideline on conducting Patients’ Experience of Care Surveys in Public Health Establishments in 2017.^13^ The guidelines highlight access to health services, medicines, patients safety, cleanliness and infection prevention and control, values and attitudes of staff, patient waiting time for care as predictors and dimensions for measuring patients’ experience with care.^13^ These dimensions are key components of patients’ experience.

The quality of healthcare in KwaZulu-Natal and in eThekwini in particular, being the largest district is, however, a concern as the performance of primary healthcare facilities in eThekwini has been suboptimal.^14^ This study therefore sought to determine the factors, which influence patients’ experiences of care in the local primary healthcare context.

## Methods

### Study design

The study was an observational cross-sectional study with an analytical component.

### Study setting

The eThekwini health district has a population of approximately 3.7 million people and is subdivided into four sub-service areas viz North, West, South Central and Lower South.^14^ eThekwini Municipality (local government) and the KwaZulu-Natal Provincial Department of Health (provincial government) provide primary healthcare services concurrently. There are 45 provincial clinics (39 primary healthcare clinics and 6 community healthcare centres) and 59 local government primary healthcare clinics. Community healthcare centres provide 24 h primary healthcare services and emergency care, have a maternity and obstetric unit, a procedure and observation room for patients for up to 48 h.

### Study population

All adult patients who received ambulatory care at six facilities during the study period (from June to November 2018) were included as part of the study population.

## Sample size and sample selection

Six primary health care facilities (three community healthcare centres and three clinics) formed the study sample. The number 6 represented 15% of the total provincial clinics and the number was selected based on access and logistics. In order to select the six sites stratified random sampling of facilities based on ideal clinic status was applied to identify three primary health care facilities that had not achieved ideal clinic status and three primary health care facilities that had achieved at least silver status in the most recent ideal clinic assessment were used to select the facilities.

The facility profile consisting of catchment population, headcount/day and service area is presented in Table 1.

**TABLE 1:** Profile of study facilities in eThekwini 2017–2018.

Facility name	Catchment population	Headcount/day	Service area
Cato Manor CHC	61 965	720	South Central
KwaMashu CHC	750 000	1419	North
Tongaat CHC	113 338	1039	North
Umbumbulu Clinic	33 353	400	Lower South
RK Khan Gateway Clinic	30 000	150	South Central

CHC, community healthcare centres.

The average outpatient headcount for adults > 18 years of age across the six facilities per month (40 000) was used as the study population. Maximum variability of 50%, 95% confidence interval with a precision of ±0.05 and six clusters were used determining a sample size of 384 participants. The proportion of patients per facility selected was based on proportion of total headcount. Systematic random sampling was used to identify patients from the six facilities identified. The average headcount/day and required sample size from each facility were used to calculate the interval.

## Data collection tool

Minor modification in terms of wording was made to the national patient experience of care (PEC) survey tool to suit the objectives of our study.^13^ The questionnaire was piloted on a sample of 20 adult patients receiving ambulatory care at one of the clinics chosen for the 2017–2018 ideal clinic cohort, who were able and willing to participate Cronbach’s alpha based on the pilot study was 0.78 indicating good internal consistency of the questions. No adjustments to the questionnaire were made, as participants in the pilot study easily understood it.

Eight socio-demographic (independent) variables (age, gender, level of education; employment status; household income; type of area of residence; marital status and self-reported health status) and the seven ministerial priority areas (access to health services, availability of medicines, patient safety, clean environment, infection prevention and control, values and attitudes of staff and acceptable patient waiting time for care) were captured through the questionnaire. The ideal clinic status level of the facilities was obtained from the ideal clinic database.

### Data collection

Questionnaires were self-administered and anonymous, however trained research assistants (post graduate students) were available to assist patients where necessary, for example, for patients who were illiterate. Research stations were set up at the exit of each facility throughout the day for 3–4 days at each facility. The questionnaire included both isiZulu and English to facilitate understanding.

### Data analysis

Stata Version 13.0 (StataCorp.2013. Stata statistical software: release 13. College Station, TX, United States: StataCorp LP) was used for data analysis. To calculate the patients’ experience score, composite scores were calculated for each of the seven priority areas, namely staff attitude, waiting time, safety and security, cleanliness, infection prevention and control, availability of basic medicines and access. The total score was then expressed as a percentage and taken to represent an overall patients’ experience score.

The patients’ experience score (> 80% regarded as good patient experience) was converted to a binary outcome variable as per convention, that is, the National Guideline on Patient Experience of Care Surveys. Bivariate analysis was first conducted to identify potentially significant associations. All variables with a significance of *p* < 0.2 were included in the multivariable logistic regression model along with ideal clinic status as it was an important variable of interest in this study.

All socio-demographic independent variables (age, gender, marital status, household income, level of education, previous visit, self-reported health status, travel time to facility > 2 h) and dependent variable (dichotomised patient experiences outcome).

Health system independent variables (ideal clinic status, type of facility) and dependent variable (dichotomised patients’ experience outcome).

## Ethical considerations

Ethical approval for the study was obtained from the University of KwaZulu-Natal Biomedical Research Ethics Committee (reference number: BE682/17) and the Provincial Health and Research Ethics Committee of the KwaZulu-Natal Department of Health. The District Manager of the eThekwini Health District provided gatekeeper’s permission. Facilities were approached for site entry via the Quality Assurance Manager at the eThekwini District Office for Health. Participants provided written informed consent after being explained the purpose of the study, the voluntary nature of participation and measures to ensure confidentiality and anonymity.

## Results

A total of 96% (369/384) of the respondents were included in the study with data being disregarded from 15 respondents because of having more than three sections incomplete.

### Socio-demographic profile of respondents

A total of 81% (299) of the respondents were female, 89.7% (331) were from the African race group and 67.2% (248) were single. A total of 123 respondents (33.3%) completed secondary school with a further 50 (13.5%) obtaining a tertiary education. A total of 268 respondents (72.6%) were unemployed. Majority of households (58.8%) had an average monthly household income of < R1600. A total of 50 (13.6%) respondents reported of their health status to be poor, whilst 47 (12.5%) reported excellent health, with the majority 72% reporting ‘good’ or ‘fair’ health. The majority of respondents (81.3%, 300) visited the same health facility in the past 12 months with 65.3% (*n* = 241) of the respondents having a travel time to the facility of less than 2 h (Table 2).

**TABLE 2:** Socio-demographic characteristics of adult patients attending primary healthcare facilities in eThekwini in 2018 (*n* = 369).

Variable	Categories	*N*	%
**Demographic characteristics**			
Gender	Female	299	81.00
	Male	60	16.30
	Missing data	10	2.70
Marital status	Single	248	67.20
	Married	74	20.00
	Widowed/divorced	22	6.00
	Living with partner	17	4.60
	Missing data	8	2.20
Race	Black	331	89.70
	Other (Indian, mixed race, white)	37	10.00
	Missing data	1	0.30
Education	No formal studies	21	5.70
	Formal education (Matric not complete)	173	46.90
	Matric	123	33.30
	College	50	13.60
	Missing data	2	0.60
Employment status	Unemployed	268	72.63
	Employed full-time + part time)	100	27.10
	Missing data	1	0.30
**Socio-economic characteristics**			
Average monthly household income	< R1600.00	217	58.80
	≥ R1600.00	115	31.20
	Missing data	37	10.00
Type of area of residence	Suburb	173	46.90
	Rural	122	33.10
	Informal (peri-urban)	71	19.30
	Missing data	3	0.80
Self-reported health status	Good	152	41.20
	Fair	115	31.20
	Poor	50	13.60
	Excellent	47	12.70
	Missing data	14	16.00
Travel time to facility > 2 h	No	241	65.30
	Yes	127	34.40
	Missing data	1	0.30

### Patients’ experience score

The median total patient experiences score for the study population was 89% (interquartile range [IQR]: 81% – 98%) (Table 2). Median scores for each patient experiences subcomponent ranged from 100% (IQR: 92% – 100%) for positive and caring attitude of staff, safety and security, cleanliness and availability of basic medicines to 75% (IQR: 50% – 100%) for infection prevention and control (Table 3).

**TABLE 3:** Median (interquartile range) of percentage scores of patient experiences and subcomponents as obtained from adult patients attending primary healthcare facilities in eThekwini in 2018.

Variable	Median	IQR
Positive and caring attitude of staff score	100	92–100
Safety and security score	100	83–100
Cleanliness score	100	100–100
Availability of basic medication score	100	80–100
Access score	93	80–100
Waiting time score	86	50–100
Infection prevention and control score	75	50–100
Total health system responsiveness score	89	81–98

IQR, interquartile range.

### Association between socio-demographic and health system factors and patients’ experience outcomes

#### Bivariate analysis

After bivariate analyses the following variables were found to have a statistically significant (*p* < 0.05): increased odds of a positive PEC, that is, good patient experiences outcome: race; income; area of residence; type of facility, signage and notices; patient service organisation; staff ID and dress code and management of patient appointments (Table 4).

**TABLE 4:** Bivariate analyses assessing the association between patient and health system factors and good patients’ experience (> 80% score).

Variable	OR	Unadjusted OR	95% CI	*p*
**Gender**				
Male	Reference	0.87	0.44–1.78	0.66
Female	0.87	Reference	0.44–1.78	0.66
**Age (years)**				
≤ 34	-	0.70	0.40–1.25	0.20
> 34	-	Reference	-	-
**Marital status**				
Single	-	1.18	0.67–2.05	0.53
Married/divorced/living with partner	-	Reference	-	-
**Race**				
Black person	-	2.54	1.16–5.43	0.008*
Indian, mixed race and white people	-	Reference	-	-
**Education**				
Lower (primary school or less)	-	1.01	0.54–1.99	0.97
Higher (high school and above)	-	Reference	-	-
**Employment status**				
Unemployed	-	1.31	0.74–2.28	0.32
Employed	-	Reference	-	-
**Income**				
< R1600.00	-	1.82	1.02–3.21	0.03*
≥ R1600.00	-	Reference	-	-
**Area of residence**				
Rural	-	4.47	2.23–9.75	< 0.001**
Urban	-	Reference	-	-
**Self-reported health status**				
Good	-	1.20	0.71–2.01	0.47
Poor	-	Reference	-	-
**Travel time**				
Travel time < 2 h	-	1.02	0.59–1.74	0.93
Travel time > 2 h	-	Reference	-	-
**Ideal clinic status**				
Gold/silver achieved	-	0.95	0.57–1.59	0.85
Status bronze or not achieved	-	Reference	-	-
**Type of facility**				
Clinic	-	5.13	2.83–9.83	< 0.001**
CHC	-	Reference	-	-
**Signage and notices**				
Signage and notices – ideal clinic dashboard score > 80%	-	3.17	1.84–5.43	< 0.001**
Signage and notices – ideal clinic dashboard score < 80%	-	Reference	-	-
**Cleanliness and hygiene**				
Cleanliness and hygiene – ideal clinic dashboard score > 80%	-	1.11	0.65–1.92	0.69
Cleanliness and hygiene – ideal clinic dashboard score < 80%	-	Reference	-	-
**Patient service organisation**				
Patient service organisation – ideal clinic dashboard score > 80%	-	2.44	1.17–4.96	0.01*
Patient service organisation – ideal clinic dashboard score < 80%	-	Reference	-	-
**Staff identity and dress code**				
**Staff identity and dress code** – ideal clinic dashboard score > 80%	-	2.96	1.72–5.07	< 0.001**
**Staff identity and dress code** – ideal clinic dashboard score < 80%	-	Reference	-	0.66
**Referral system**				
**Referral system** – ideal clinic dashboard score > 80%	-	0.46	0.27–0.78	< 0.001**
**Referral system** – ideal clinic dashboard score < 80%	-	Reference	-	-
**Management of patient appointments**				
**Management of patient appointments** – ideal clinic dashboard score > 80%	-	3.23	1.89–5.57	< 0.001**
**Management of patient appointments** – ideal clinic dashboard score < 80%	-	Reference	-	-
**Security**				
Security-ideal clinic dashboard score > 80%	-	0.39	0.21–0.74	< 0.001**
Security-ideal clinic dashboard score < 80%	-	Reference	-	-

OR, odds ratio; CI, confidence interval; CHC, community healthcare centre.

* , Significance of *p* < 0.05;

** , significant of *p* < 0.001.

Patients who resided in rural areas reported a 4.47 times greater odds of good patients’ experience compared with patients who resided in urban areas (95%) (CI 2.23–9.75). The odds of good patients’ experience score were 2.54 times greater if the patient was black (95% confidence interval [CI]: 1.16–5.43). Patients who had a monthly household income < R1600.00 had a 1.82 times increased odds of reporting good patient experiences score compared with patients whose average monthly household income was ≥ R1600.00 (95% CI: 1.20–3.21). No significant associations were observed for the socio-demographic variables age, gender, marital status, level of education, employment, self-reported health status and previous visit or travel time > 2 h (Table 3).

Patients who attended clinics had a 5.13 times increased odds of reporting good patient experiences score compared with patients who attended community healthcare centres (95% CI: 2.83–9.63). Patients who attended primary healthcare clinics and scored 80% or above for management of patient appointments, per the ideal clinic assessment system, had a 3.23 times increased odds of reporting good patient experiences score compared with those who attended primary healthcare clinics and scored less than 80% (95% CI: 1.89–5.57).

Similarly, patients who attended primary healthcare clinics and scored 80% or above for signage and notices, per the ideal clinic assessment system, had a 3.17 times increased odds of reporting good health systems responsiveness than those who attended primary healthcare clinics that scored < 80% (95% CI: 1.84–5.43). The odds of reporting good patients’ experience score were 2.96 times higher amongst patients who attended primary healthcare clinics that scored 80% and above for staff identification and dress code, per the ideal clinic assessment system, compared with those who attended primary healthcare clinics and scored less than 80% (95% CI: 1.72–5.07). Notably, there was no association between overall ideal clinic status and good patients’ experience score (OR: 0.95; 95% CI: 0.57–1.59) (Table 3).

#### Multivariate analysis

In addition to ideal clinic status that was the key variable of interest, all variables with a significance level of *p* < 0.2 were included in the multivariable model. Thereafter, variables were assessed for collinearity and those who were found to be collinear were excluded.

The strongest association was found for type of facility for which the odds ratio increased by > 10% from 5.13 in bivariate analysis to 6.53 (95% CI: 2.76–15.48). This implied that there was negative confounding by the remaining variables. There was a greater than 10% increase in the odds ratio for ideal clinic status, which also had a positive association with patients’ experience score in the multivariate analysis (adjusted odds ratio [AOR]: 1.75; 95% CI: 0.92–3.35) although only significant at the 0.1 level. There was therefore negative confounding by the remaining variables (Table 5).

**TABLE 5:** Multivariate analyses assessing the association between patient and health system factors and good patients’ experience (> 80% patients’ experience score).

Variable	Adjusted OR	95% CI	*p*
**Race**			
Black	2.12	0.86–5.26	0.104
Indian, mixed race and white	Reference	-	-
**Income**			
< R1600	1.32	0.74–5.23	0.355
≥ R1600	Reference	-	-
**Area of residence**			
Rural	1.23	0.48–3.15	0.662
Urban	Reference	-	-
**Ideal clinic status**			
Gold/silver achieved	1.75	0.92–3.35	0.090*
Status bronze or not achieved	Reference	-	-
**Type of facility**			
Clinic	6.53	2.76–15.48	0.001**
CHC	Reference	-	-

OR, odds ratio; CI, confidence interval; CHC, community healthcare centre.

* , Significance of *p* < 0.05;

** , significant of *p* < 0.001.

None of the socio-demographic factors was significantly associated with patients’ experience in this analysis. The odds ratio for area of residence decreased by > 10% (from 4.47 to 1.23), which implies that there was positive confounding by the remaining variables.

## Discussion

None of the socio-demographic variables were significantly associated with patients’ experiences in this study, which is consistent with the 2009 South African study.^15^ This differs from other previous studies.^16,17,18^ The lack of association between household income and patients’ experience may be explained by the similar access to healthcare services amongst all respondents in the different income categories in this study. Studies that had previously reported better patient satisfaction in higher income groups compared high-income with low-income groups and attributed the difference in satisfaction to differential access to public versus private care.^16,18^ This suggests that it is the type of provider that mediates the association between income and patients’ experience.^16^ The homogeneity in the study population, being largely, unemployed, female, single and of the African race group also limited the comparisons that could be made. Similarly most patients reported a previous visit in the past 12 months, which also limited the investigation of this variable.

In this study patients who attended clinics were more likely to have reported good patients’ experience compared with those who had attended community healthcare centres. It has been consistently observed that smaller facilities report higher levels of patient satisfaction.^19,20,21^ A 2010 study in the general practice setting in the United Kingdom reported better patient satisfaction with access to care at smaller practices compared with larger practices.^19^ Similarly, smaller hospitals reported better patient satisfaction in a study in United States in 2017.^20^ In China where primary healthcare clinics services can be accessed at primary, secondary and tertiary levels of care, better patient satisfaction was reported amongst community healthcare centres (smaller facilities) compared with hospitals (larger facilities).^21^ There are many possible reasons for this, which include patients’ perception of the environment in larger facilities as busier and more difficult to navigate.^20^ Health system level factors that are known to vary between clinics and community healthcare centres, for example, workload, staffing and effective management are some of the possible confounding factors, which warrant further exploration.

The following variables: signage and notices, patient service organisation, staff ID and dress code and management of patient appointments that form part of component 3 of the ICRM framework showed significance for positive patients’ experience at a bivariate level. The positive association from amongst these factors support the concept that a patients’ experience at facility that attains ideal clinic status is much better.

Ideal clinic status was also associated with good patients’ experience although this was a much weaker association. A 2017 nationally representative study in South Africa found that positive PEC was only significantly higher amongst the best performing ideal clinic facilities.^22^

A similar positive association between a quality of care measure and patient satisfaction with access to primary healthcare clinics was observed in the 2010 United Kingdom study, which showed that a 10 point increase in quality score was associated with a higher patient satisfaction score of up to 3.4%.^19^ In contrast a 2011 study in Germany in the hospital setting, showed no association between patient satisfaction (as measured by willingness to recommend the hospital to family) and hospital accreditation status (OR: 0.98; 95% CI: 0.84–1.13).^23^

Although all necessary steps to maintain the integrity of the study were complied with, it was found that 15 respondents did not fully complete the questionnaire for various reasons, which resulted in their exclusion, thus introducing selection bias. The measurement instrument used in both this study is not weighted in term of importance that patients assign to the dimensions of patients’ experience measured. It is possible that there was an overestimation or underestimation of good patients’ experience. This would however have been non-differential misclassification, which may have biased results towards the null. The measures of health system level factors in this study relied on secondary data obtained from the ideal clinic database. The accuracy of this data is therefore limited. Efforts were however made to reduce misclassification of exposure by using the most objective measure, that is, peer review assessments rather than self-assessments or district office assessments.

Social desirability and gratitude bias where patients may possibly over report positive experiences may have resulted in an overestimation of good patients’ experience.

The type of facility that patients had attended created a false association between area of residence and patients’ experience. This may have been because of rural residents being more likely to access clinics and clinics being associated with better patients’ experience in the study. Firstly, historical lack of facilities make rural residents more appreciative of clinics. Secondly, the type of facility masked the effect of ideal clinic status on patients’ experience. This may have been because there being more community healthcare centres that had achieved ideal clinic status in the study (two community healthcare centres versus 1 clinic) and community healthcare centres being associated with poor patients’ experience in this study. When the effect of type of facility was controlled for, ideal clinic status was positively associated with patients’ experience, however, the association was still not significant.

Ideal clinic status increased the effect size of the positive association between type of facility and good patient-reported patients’ experience. This implies that whilst ideal clinic status achievement alone was not sufficient to significantly influence good patients’ experience, its value in specific contexts varies. The association was stronger in the context of clinics.

The study was a quantitative study and did not collect qualitative information on patients’ preferences and values.

## Conclusion

Patients attending clinics had a better patient experience compared with community health centres. Although ideal clinic status showed a positive association with good patient experiences this did not reach statistical significance. This may suggest that factors other than structural improvements play an important role in patients’ experience. Ideal clinic improvements may have greater value in certain contexts compared with others. Furthermore qualitative studies are required to explore patients’ values and preferences before further investment in upgrading and re-organising facilities to achieve ideal clinic status are implemented.
